# Supplementary material for: The causal relationship between the human gut microbiota and pyogenic arthritis: a Mendelian randomization study
Source: Front Cell Infect Microbiol. 2024 Nov 26;14:1452480. doi: 10.3389/fcimb.2024.1452480 (PMC11629706; doi:10.3389/fcimb.2024.1452480)
Supplement: Supplementary file 1 [file DataSheet1.zip › mendelian test/ebi-a-GCST90027612.csv_forest.pdf]

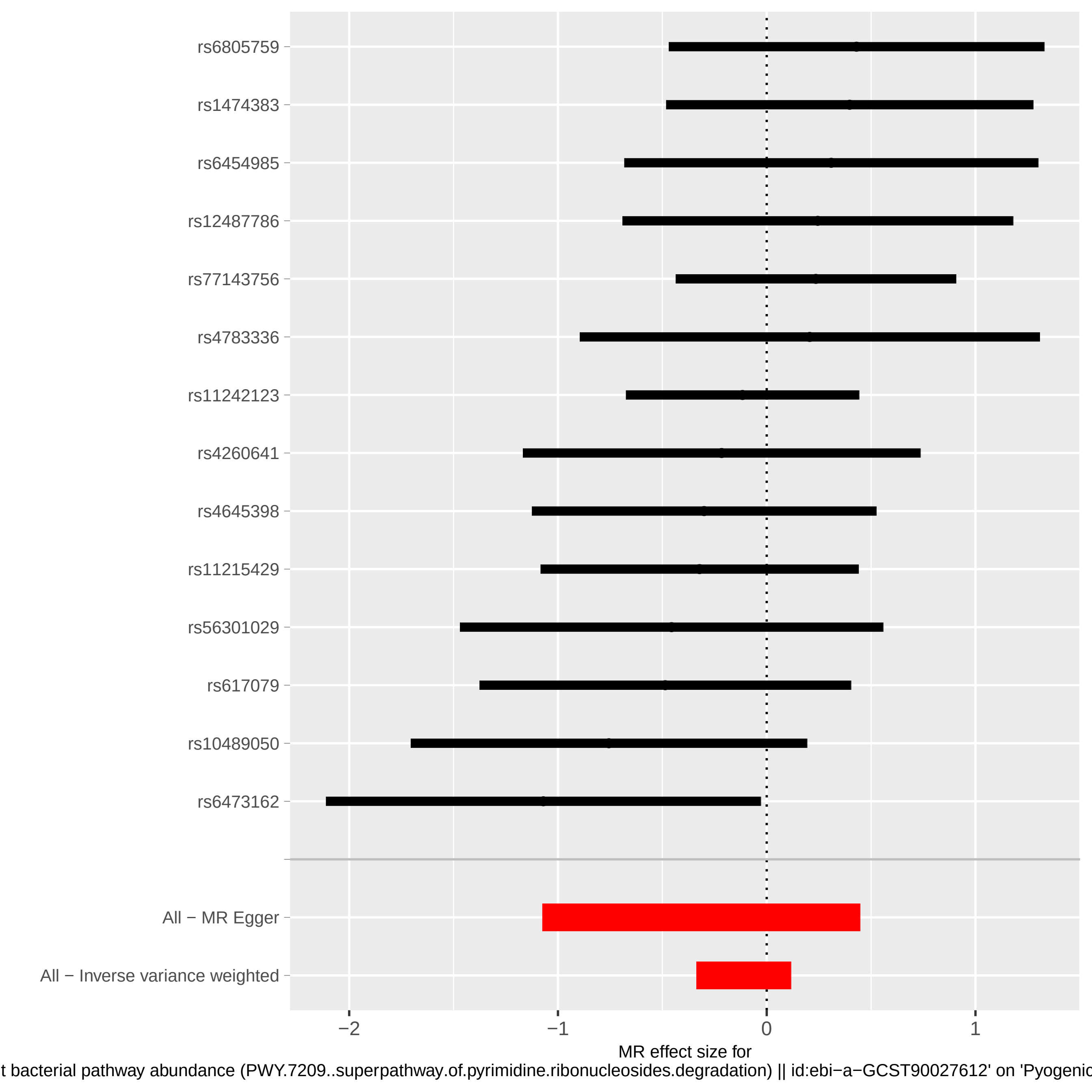

rs6805759
rs1474383
rs6454985
rs12487786
rs77143756
rs4783336
rs11242123
rs4260641
rs4645398
rs11215429
rs56301029
rs617079
rs10489050
rs6473162
All − MR Egger
All − Inverse variance weighted
−2
−1
0
1
MR effect size for
t bacterial pathway abundance (PWY.7209..superpathway.of.pyrimidine.ribonucleosides.degradation) || id:ebi−a−GCST90027612' on 'Pyogenic
